# Viscoelastic Cell Mechanics and Actin Remodelling Are Dependent on the Rate of Applied Pressure

**DOI:** 10.1371/journal.pone.0043938

**Published:** 2012-09-11

**Authors:** Priyanka Pravincumar, Dan L. Bader, Martin M. Knight

**Affiliations:** Institute of Bioengineering, School of Engineering and Material Science, Queen Mary University of London, London, United Kingdom; Emory University/Georgia Insititute of Technology, United States of America

## Abstract

**Background:**

Living cells are subjected to external and internal mechanical stresses. The effects of these stresses on the deformation and subsequent biological response of the cells remains unclear. This study tested the hypothesis that the rate at which pressure (or stress) is applied influence the viscoelastic properties of the cell associated with differences in the dynamics of the actin cytoskeleton.

**Principal Finding:**

Micropipette aspiration was used to determine the instantaneous and equilibrium moduli and the viscosity of isolated chondrocytes based on the standard linear solid (SLS) model and a variation of this incorporating Boltzmann superposition. Cells were visualised for 180 seconds following aspiration to 7 cmH_2_O at 0.35, 0.70 and 5.48 cmH_2_O/sec. Cell recovery was then examined for a further 180 seconds once the pressure had been removed. Reducing the rate of application of pressure reduced the levels of cell deformation and recovery associated with a significant increase in modulus and viscosity. Using GFP transfection and confocal microscopy, we show that chondrocyte deformation involves distortion, disassembly and subsequent reassembly of the cortical actin cytoskeleton. At faster pressure rates, cell deformation produced an increase in cell volume associated with membrane bleb formation. GFP-actin transfection inhibited the pressure rate dependent variation in cell mechanics indicating that this behaviour is regulated by GFP-sensitive actin dynamics.

**Conclusion:**

We suggest that slower rates of aspiration pressure enable greater levels of cortical actin distortion. This is partially inhibited by GFP or faster aspiration rates leading to membrane bleb formation and an increase in cell volume. Thus the rate of application of pressure regulates the viscoelastic mechanical properties of living cells through pressure rate sensitive differences in actin dynamics. Therefore cells appear softer when aspirated at a faster rate in contrast to what is expected of a normal viscoelastic material.

## Introduction

Living cells in a wide variety of tissues are subjected to a complex mechanical loading environment comprising of both externally applied and internally generated mechanical forces. It is increasingly clear that this loading regulates gene expression and many aspects of cells function. In chondrocytes within articular cartilage, mechanical loading regulates the synthesis and turnover of extracellular matrix molecules and is therefore essential for the health and homeostasis of the tissue [1], [2]. The underlying mechanotransduction processes through which chondrocytes and other cell types, sense and response to mechanical stimuli are unclear [3]. It is however increasingly apparent that the mechanotransduction response is influenced by the rate at which the mechanical load is applied [4].

The actin cytoskeleton provides a degree of mechanical integrity to cells and is therefore involved in the regulation of cell biomechanics, deformability and mechanotransduction [5], [6]. In articular chondrocytes, the actin microfilaments form a cortical mesh beneath the cell membrane. The actin cytoskeleton exists in a constant state of turnover between polymerised filamentous F-actin and globular G-actin. This facilitates the remodelling of structures such as lamelipodia, filopodia and stress fibres and is important in many aspects of cell function including motility, morphogenesis, cell cycle progression and differentiation. In addition the organisation of the actin cytoskeleton is influenced by various types of mechanical loading including compression [7], tension [8] hydrostatic pressure [9] and osmotic challenge [10]. Mechanical loading also influences actin organisation in purified actin preparations [11]–[14] and isolated actin stress fibres [15], [16]. Recent studies have shown that purified actin experiences strain hardening and softening in response to mechanical perturbation [11], [12]. However it is unclear whether such phenomena also exist within the cell.

This study uses micropipette aspiration to measure the biomechanics of isolated chondrocytes in order to test the hypothesis that the cellular mechanical properties are dependent on the rate of application of applied pressure. Actin dynamics are visualised using a GFP tag which reveals cortical actin distortion, breakdown and subsequent remodelling which underpins cell deformation. Thus this study reveals for the first time that the rate at which pressure is applied influences cellular viscoelastic behaviour and that this occurs through pressure rate dependent differences in actin dynamics.

## Results

### Chondrocyte viscoelastic behaviour is regulated by the rate of applied pressure

**Figure 1:**
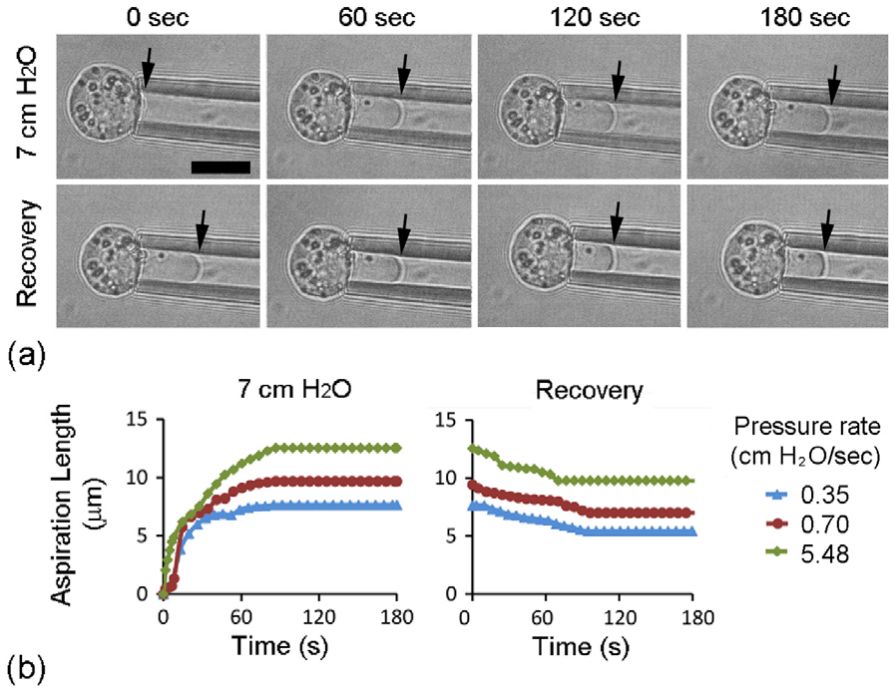
Isolated chondrocytes exhibit viscoelastic behaviour during micropipette aspiration. a) Representative brightfield images showing micropipette aspiration of a chondrocyte subjected to a 7 cmH_2_O aspiration pressure (top row) and during a the subsequent recovery period at 0 c mH_2_O (2^nd^ row). Pressure was applied and removed at an aspiration rate 0.35 cmH_2_O/sec. Arrows indicate the extent of the aspiration length into the micropipette. Scale bar represents 10 microns. b) Temporal changes in aspiration length showing characteristics viscoelastic behaviour during aspiration and recovery for individual chondrocytes subjected to aspiration applied at 0.35, 0.70 and 5.48 cmH_2_O/sec. Time zero starts the moment the aspiration pressure is applied which in the case of the slowest pressure rate occurs 20 seconds before the pressure reaches the equilibrium value of 7 cmH_2_O.

**Figure 2:**
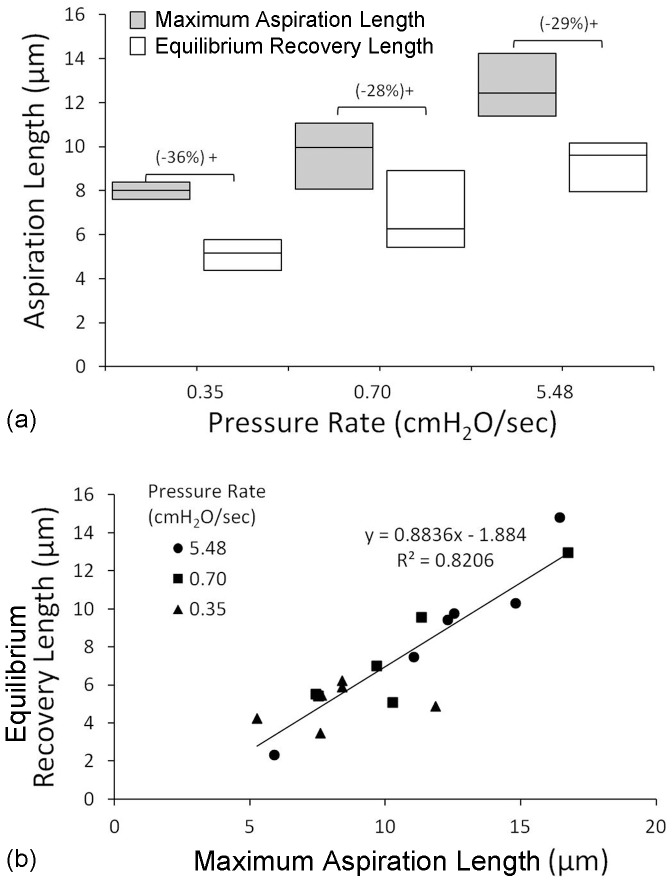
The aspiration rate influences the maximum aspiration length and subsequent equilibrium recovery length as well as the increase in cell volume that occurs during aspiration. a) Median and quartiles values for cells subjected to 7 cmH_2_O aspiration pressure applied at 0.35, 0.70 and 5.48 cmH_2_O/sec. All cells showed a statistically significant reduction in aspiration length during the recovery period (+, p<0.05). The median percentage recovery in aspiration length is given in parentheses. b) There was a linear relationship between the maximum aspiration length at 7 cmH_2_O and the equilibrium recovery length at 0 cmH_2_O.

Using the micropipette aspiration technique, the effect of loading rate on chondrocyte mechanics was examined. An aspiration pressure of 7 cmH_2_O was applied at a rate of either 0.35, 0.70 or 5.48 cmH_2_O/sec. The viscoelastic deformation of individual cells was monitored at 7 cmH_2_O for 180 sec, followed by further 180 sec upon removal of pressure (Fig. 1a). At each of the three different pressure rates, chondrocytes initially aspirated rapidly into the micropipette followed by a decreased rate of aspiration until an equilibrium was reached. This was quantified by the temporal change in aspiration length as shown for representative cells in Figure 1b. After the aspiration pressure was removed, cells exhibited a partial recovery reaching an equilibrium recovery length which was approximately 30% of the peak aspiration length (Fig. 2a). Close examination of the data for individual cells revealed that the equilibrium recovery length was proportional to the maximum aspiration length as indicated by the linear model in figure 2b. Both the maximum aspiration and equilibrium recovery lengths were greater when the pressure was applied more rapidly, the differences between the three pressure rates being statistically significant. At the slower aspiration rate, cell deformation occurred with no statistically significant change in cell volume but a significant increase in surface area (16%). By contrast, at the faster aspiration rates cell deformation was associated with a significant increase in cell volume by approximately 5% and a 20% increase in surface area.

**Figure 3:**
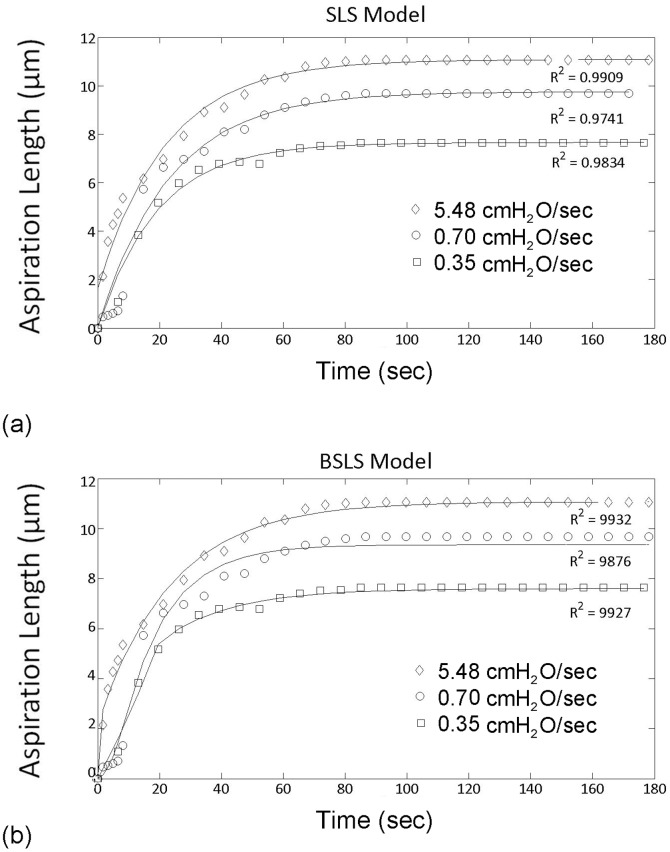
Viscoelastic cell deformation can be modelled using the SLS and BSLS models. Representative examples of cell aspiration data fitted using a) the SLS model and b) the BSLS model. Cells were aspirated at 0.35, 0.70 and 5.48 cmH_2_O/sec.

The temporal change in aspiration length during initial 180 second period was mathematically fitted, using either the Standard Linear Solid (SLS) or the Boltzmann Standard Linear Solid (BSLS) models (Fig. 3). These models yielded the three viscoelastic parameters, namely, instantaneous modulus, equilibrium modulus and viscosity. Cells were rejected from analysis if they did not aspirate, if the analytical model did not converge or produce an accurate fit (R<0.95) or if the estimated constants reached the preset limit (k_2_ = 100). Table 1 shows the number of cells rejected under each of the above criteria and hence the total number successfully analysed for each condition.

**Table 1:** The numbers of cells analysed for each experimental condition including the numbers of cells rejected from the analysis under different criteria.

Pressure Rate (cmH_2_O/sec)	Total No. of Cells Tested	No. Aspiration	No. for which model converged		No. for which model produced R≥0.95		No. for which k_2_<100 *Total analysed*	
			SLS	BSLS	SLS	BSLS	SLS	BSLS
**Non-Transfected Chondrocytes**								
5.48	26	23	23 (100%)	23 (100%)	22 (96%)	23 (100%)	21 (95%)	20 (87%)
0.70	16	16	16 (100%)	16 (100%)	14 (94%)	15 (94%)	12 (80%)	13 (87%)
0.35	16	16	16 (100%)	16 (100%)	15 (94%)	16 (100%)	10 (67%)	14 (88%)
**Transfected Chondrocytes**								
5.48	12	11	N/A	11 (100%)	N/A	11 (100%)	N/A	10 (91%)
0.35	19	16	N/A	16 (100%)	N/A	15 (94%)	N/A	13 (87%)

Values in parentheses indicate the number of cells that passed each successive exclusion criteria as a percentage of the total number of aspirated cells.

**Figure 4:**
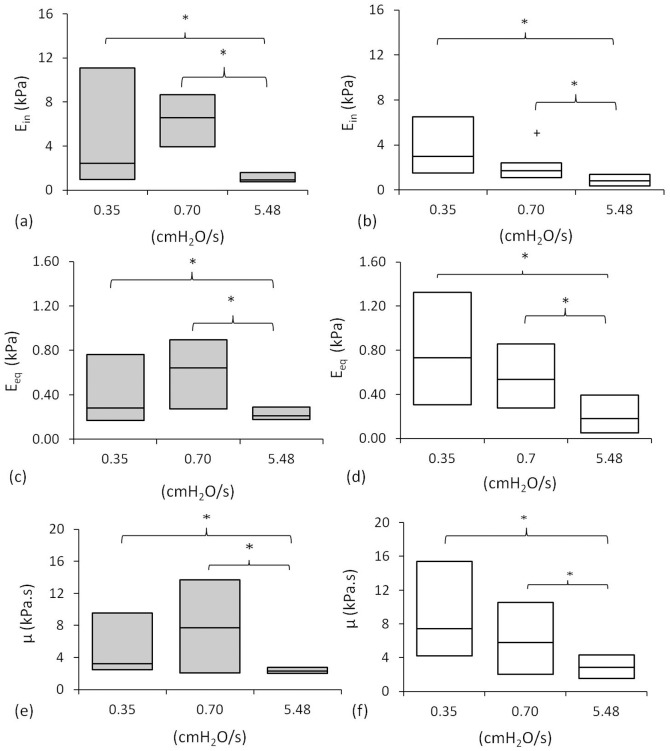
Pressure rate influences cellular mechanical properties estimated using the SLS and BSLS models. Plots show median and quartile values for instantaneous modulus (a,b), equilibrium modulus (c,d) and viscosity (e,f) estimated using the SLS model (a,c,e) and BSLS model (b,d,f) for cells aspirated at 0.35, 0.70 and 5.48 cmH_2_O/sec. Statistically significant differences (p<0.05) are indicated between the ramp rates (*) and between the models (+).

Both the SLS and BSLS models produced similar values for the equilibrium modulus and the viscosity at each aspiration rate. However, at aspiration rates of 0.35 and 0.7 cmH_2_O/sec, the SLS model tended to over-estimate the instantaneous modulus compared to the value estimated using the BSLS model (Fig. 4a). The SLS model was also less able to fit the data at the slower aspiration rates as shown by the higher percentage of cells rejected from analysis due to the value of k_2_ reaching the present limit (Table 1). This reflects the fact that the SLS model does not account for the finite rate at which aspiration pressure is applied and is therefore less suitable for modelling slow aspiration rate data. Never-the-less, both models show an increase in the moduli and the viscosity with decreasing aspiration rate, the differences being statistically significant as shown in figure 4. Thus for example, the BSLS analysis revealed that for cells tested with applied aspiration rates of 5.48 cmH_2_O/sec there was a 3.6 fold increase in instantaneous modulus and a 4.1 fold increase in equilibrium modulus compared to values at a slower aspiration rate of 0.35 cmH_2_O/sec. This inverse relationship between aspiration rate and modulus is the opposite of what would be expected for a normal viscoelastic material.

### Cell deformation involves actin distortion, breakdown and remodelling

**Figure 5:**
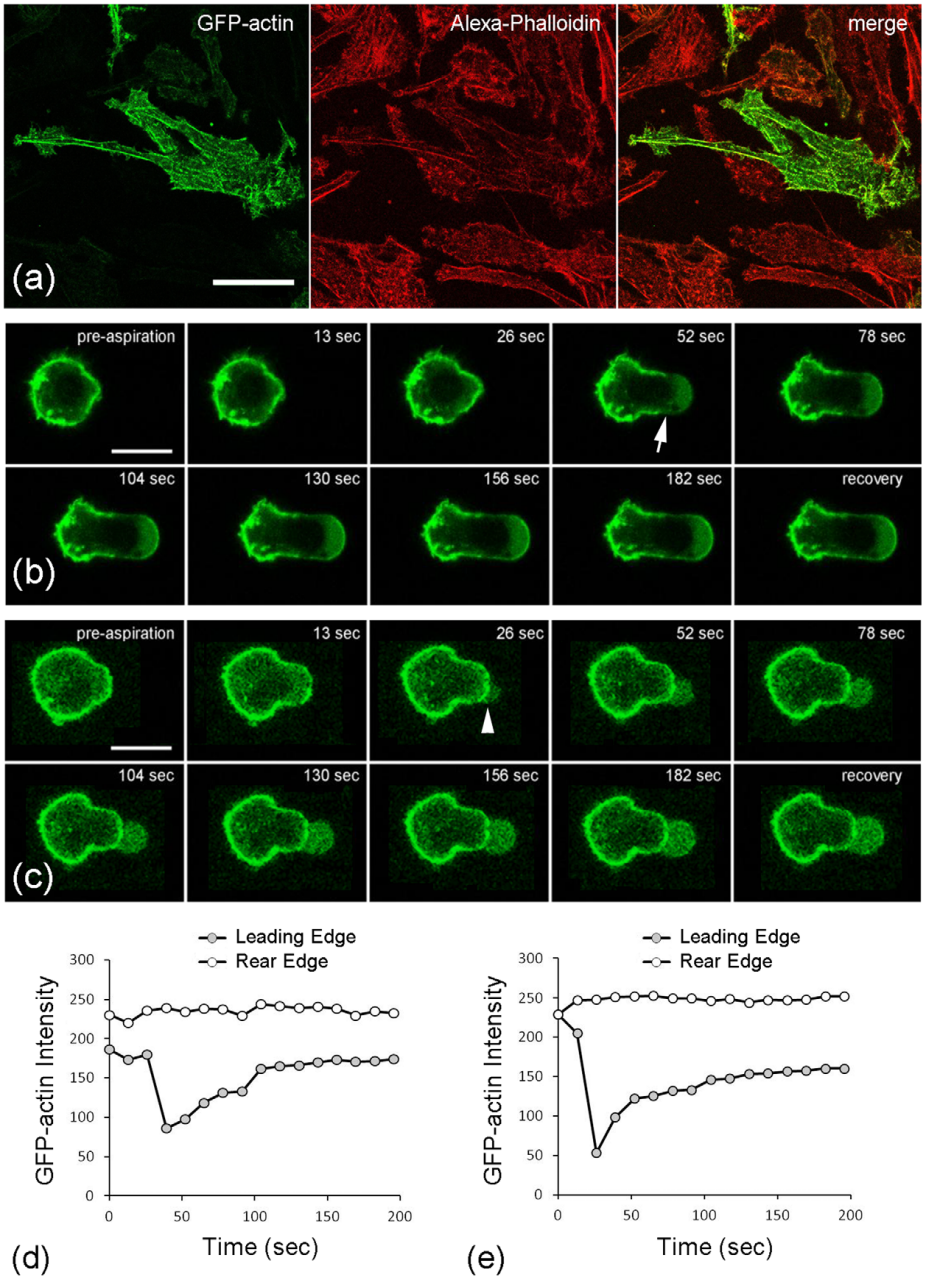
Cell deformation is associated with actin distortion, disassembly and remodelling. a) Representative confocal images showing characteristic actin organisation in a chondrocyte cultured in monolayer and transfected with GFP-actin (green). F-actin has been co-labelled with alexa-phalloidin (red). Selected images from time series showing GFP-actin distortion and dynamics during micropipette aspiration applied at a) 0.35 and b) 5.48 cmH_2_O/sec. Arrow indicates breakdown/fluidization of the actin cortex. Arrowhead shows initiation of a membrane bleb. All scale bars represent 10 microns. The corresponding temporal changes in GFP actin intensity at the leading edge (ROI 1) and the rear edge (ROI 2) are shown for a cell aspirated at d) 0.35 and e) 5.48 cmH_2_O/sec.

Primary chondrocytes were successfully transfected with GFP-actin and showed actin organisation similar to that seen in non-transfected cells stained with AlexA-Phalloidin 555 (Fig. 5a–c). In both cases, chondrocytes in monolayer displayed multiple thin stress fibers. Representative images of GFP-actin transfected chondrocytes subjected to aspiration pressure rate of 0.35 cmH_2_O/sec are shown in figure 5b. In a similar manner to non-transfected chondrocytes, GFP-actin transfected cells exhibited a viscoelastic response during micropipette aspiration. The temporal change in aspiration lengths measured from the GFP-actin images matched that measured from the brighfield images obtained from the same cell.

Examination of the GFP-actin images shows that chondrocytes deformation during aspiration was associated with distortion and remodelling of the cortical actin into the micropipette leading. GFP-actin intensity was quantified within specific regions of interest (ROIs) positioned over the leading edge of the cell in the micropipette (ROI1) and the cell cortex outside of the micropipette (ROI2). These ROI positions therefore change as the cell is aspirated. In the majority of cells, the actin cortex at the leading edge broke down and then remodelled as the cell continued to deform into the micropipette. This behaviour is shown in figure 5b for a representative cell aspirated at 0.35 cmH_2_O/s. For some cells, particularly those cells aspirated at the faster rate of 5.48 cmH_2_O/s, the actin cortex in the micropipette appeared to remain intact but a membrane bleb appeared which then aspirated further into the micropipette as shown in figure 5c. Even in these cells it is possible that localised cortical actin breakdown may have occurred out of the plane of focus enabling formation of the membrane bleb. The corresponding temporal changes in intensity within ROI1 clearly shows the actin breakdown and/or bleb formation which occurred between 25 and 50 sec after the application of pressure and at a mean aspiration length of approximately 5 µm into the micropipette. In all cases, the cortical actin breakdown or bleb formation was followed by a gradual remodelling of the cortical actin at the leading edge (ROI 1). The GFP-actin intensity in ROI 2, representing a non-aspirated region of the cell outside of the micropipette, remained stable throughout the experiment indicating the spatial localisation of the actin dynamics.

### GFP-sensitive actin dynamics governs cellular response to the rate of applied pressure

**Figure 6:**
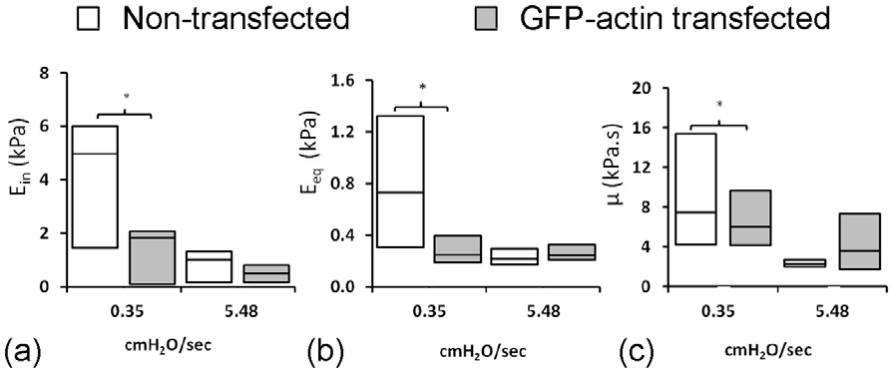
GFP-actin transfection inhibits pressure-rate sensitive changes in cell mechanics. The BSLS model was used to estimate the mechanical properties of transfected and non-transfected chondrocytes aspirated at two different pressure rates of 0.35 and 5.48 cmH_2_O/sec. Media and quartile values are shown for a) instantaneous modulus b) equilibrium modulus and c) viscosity. Statistically significant difference exist between transfected and non-transfected chondrocytes at 0.35 cmH_2_O/sec (*, p<0.05). For non transfected cells the moduli and viscosity were significantly greater at the slower aspiration rate (p<0.05).

Transfected chondrocytes were aspirated at two different pressure rates of 0.35 and 5.48 cmH_2_O/sec and the mechanical parameters estimated using the BSLS model. The cells expressing GFP-actin exhibited similar mechanical properties to non-transfected cells when aspirated at a rate of 5.48 cmH_2_O/sec, such that there were no significant differences between the two groups in terms of the three viscoelastic parameters (Fig. 6). However the transfected cells did not exhibit the increased moduli and viscosity observed at the slower aspiration rate of 0.35 cmH_2_O/sec. Thus at 0.35 cmH_2_O/sec, the non transfected cells exhibited higher values for both instantaneous and equilibrium moduli and viscosity when compared to the non-transfected cells, the differences being statistically significant (Fig. 6).

## Discussion

The present study tested the hypothesis that the rate at which pressure is applied influences the viscoelastic properties of isolated chondrocytes and that this involves modulation of actin dynamics. Isolated bovine articular chondrocytes were successfully aspirated using a micropipette system incorporating a computer controlled pump, which provided temporal control over the height of fluid in a reservoir and hence the rate at which aspiration pressure was applied. The chondrocytes showed characteristic viscoelastic behaviour, such that the application of a static pressure induced a gradual increase in aspiration length into the micropipette, reaching equilibrium after approximately 90 seconds (Fig. 1).

This behaviour was successfully modelled by the Standard Linear Solid (SLS) model in agreement with previous studies [17]. In addition the data was also modelled using the Boltzmann Standard Linear Solid (BSLS) model, which is a modification of the SLS incorporating Boltzmann superposition to account for a finite rate at which pressure is applied [18]. This model thereby provides an improved approximation of the cellular mechanical properties, particularly at slower ramp rates where the time over which pressure is applied is greater than 2 seconds [19]. This explains why when using the SLS model for analysing slow aspiration rate data, the instantaneous modulus values tend to be larger than those estimated using the BSLS model (Fig. 3) and there are a relatively high percentage of cells rejected due to k_2_ reaching the preset limit (Table 1). However, both models indicate that the rate at which pressure is applied influences cellular viscoelastic behaviour. At the fastest aspiration rate of 5.43 cm H_2_O/sec, the moduli and viscosity agreed with previous estimates made under similar conditions, with an equilibrium or Young's modulus of 0.22 kPa [9], [10], [17]–[30]. However, at low aspiration rates, the level of cell deformation into the micropipette was reduced corresponding to higher values of modulus and viscosity (Figs. 2 and 3). This pressure rate phenomena cannot be explained by the viscoelastic behaviour of the cell, but instead suggests that structural changes or remodelling are occurring producing a change in the apparent mechanical properties of the cell.

It is well established that the actin cytoskeleton provides a major contribution to the mechanical properties of living cells [9], [31], [32]. Thus, the present findings demonstrating a reduction in cell modulus associated with increased pressure rate, may be due to alterations in actin dynamics. Aspiration of chondrocytes expressing GFP-actin revealed that cell deformation during micropipette aspiration was associated with distortion of the cortical actin, frequently leading to a sudden breakdown of the actin cortex within the micropipette. This was followed by formation of a membrane bleb and then a gradual remodelling of the cortical actin within the bleb (Fig. 5). The timescales of rapid actin fluidization and slower reassembly over approximately 5 minutes, agrees well with previous studies of actin dynamics and disassembly in response to mechanical stimuli [16], [33]. Studies with smooth muscle cells cultured on elastic substrates, demonstrated rapid actin fluidization in response to substrate stretch [33]. These studies found that the fluidization occurred through the release of tensile forces driving actin disassociation, rather than activation of cellular signalling pathways. Such finding may explain why the cortical actin breakdown observed in the present micropipette aspiration study was more pronounced than that previously reported in compressed chondrocytes. The breakdown in cortical actin may therefore occur due to physical detachment of the cell membrane and a resulting loss of cortical tension. Interestingly this rapid disappearance of the cortical actin and/or the formation of a membrane bleb was not associated with any step change in aspiration length. We suggest that the loss of tension associated with actin breakdown and bleb formation allows the inherent cellular osmotic swelling pressure to drive fluid intake leading to a small increase in total cell volume as the membrane bleb expands into the micropipette (Fig. 5). Actin remodelling behaviour has also been reported in a carcinoma cell line, such that micropipette aspiration induced cortical actin breakdown followed by bleb formation and subsequent actin remodelling [34]–[36]. Indeed, physiological bleb formation is important in cell locomotion [37], mitosis [38], [39] and apoptosis [39]. However, the present study is the first to associate this actin behaviour and bleb formation with the cellular viscoelastic biomechanics and the influence of pressure rate.

The faster aspiration rate induces an increase in cell volume during deformation. This suggests that there is greater physical detachment of the cell membrane from the cortical actin and hence increased bleb formation leading to the reduction in apparent cell moduli. This may be associated with pressure rate dependent behaviour of the actin such that at faster aspiration rates there is a decreased ability of the actin to distort, remodel and flow into the micropipette. Such behaviour appears to be inhibited by the presence of the GFP tag in agreement with previous studies which report that although GFP does not change actin organisation as shown here (Fig. 4a), it can influence actin dynamics [40], [41]. Hence this could explain why cells transfected with GFP-actin did not show the increased moduli at slower aspiration rates (Fig. 6) as observed in non transfected cells (Fig. 4). Thus inhibition of the pressure-rate sensitive effects on cellular mechanics in cells transfected with GFP-actin, supports the hypothesis that this behaviour is regulated by GFP-sensitive actin dynamics although the precise mechanism is still unclear.

The incomplete recovery of the cell morphology upon removal of pressure also indicates a degree of actin structural remodelling (Fig. 1). The level of permanent deformation was proportional to the equilibrium aspiration length at 7 cm H_2_O (Fig. 2). Extrapolation of the linear model fitted to these data suggests that permanent deformation occurs if the cell is aspirated to approximately 2 µm or more (Fig. 2b). Furthermore, the present data indicates that the development of permanent deformation is enhanced at faster aspiration rates and is linked to increased bleb formation and the fluidisation and remodelling of the cortical actin cytoskeleton.

In summary, our data indicates that cell deformation during micropipette aspiration results in disassembly and subsequent reassembly of specific areas of the cortical actin with associated bleb formation and increase in cell volume. We suggest that this behaviour is sensitive to the rate of applied pressure. Hence slower aspiration rates allow sufficient time for mechanically induced actin remodelling thereby preventing membrane detachment and bleb formation. This results in increased cell modulus and viscosity and less permanent deformation at slower rates of aspiration. This study therefore reveals for the first time, that the rate at which pressure is applied regulates the viscoelastic mechanical properties of living cells and that this is related to the finite time taken for the actin cytoskeleton to remodel and distort during cell deformation. These findings are important in understanding how cellular biomechanics, the actin cytoskeleton and membrane bleb formation are regulated by external and internal mechanical forces.

## Materials and Methods

### Chondrocyte Isolation

The front feet from bovine steers were obtained from a local abattoir (C. Humphreys and Sons, Essex). Full depth cartilage was removed from the proximal articular surfaces of the metacarpalphalangeal joints. Cartilage slices were immersed in Dulbecco's minimal essential medium supplemented with 10% (v/v) foetal calf serum (DMEM+10% FCS, Gibco, Paisley, UK). The diced cartilage slices were incubated on a roller mixer at 37°C/5% CO_2_ for 1 hour in pronase (Type E, 700 units/mL. BDH Industries Ltd, Poole, UK) followed by 16 hours in 30 mL collagenase (Type XI, 100 units/mL, Sigma-Aldrich, Poole, UK). The digest was filtered through a 70 µm pore size sieve (Falcon, Oxford,UK) and the resulting cell suspension washed twice in DMEM+10% FCS.

### GFP-actin transfection

Primary chondrocytes were transduced with GFP-actin using BacMam reagent (Invotrogen, CellLight® Reagent BacMam 2.0). The reagent was directly added to cell suspension with cell density of 2×10^6^ cells/mL at 1–10% vol/vol. The cells were incubated for 2 hr at room temperature on a rocker. The cells were then plated at 0.5×10^6^ cells/cm^2^ in a 96 well plate and incubated at 37°C and 5% CO_2_ for 16–19 hrs. The reagent was diluted by the addition of fresh DMEM+10% FCS (100 µL/well) and incubated overnight at 37°C and 5% CO_2_. Cells were trypsinised and used for the micropipette aspiration experiments.

### Micropipette Aspiration

Micropipette aspiration, similar to that previously described [31], [42], [43], was performed to determine the viscoelastic properties of individual chondrocytes. The micropipette aspiration system was used in conjunction with a confocal laser scanning micropipette (SP2, Leica, UK). The micropipettes were constructed from glass capillaries (G-1, Narshinghe Intentional, UK) that were pulled by a commercial system (P97, Sutter Instrument, CA, USA) and fractured to an inner diameter of approximately 6.5–7.5 µm. The micropipettes were coated with silicone solution (Sigmacote, Sigma, MO, USA) to prevent cell adhesion

The cell suspension was placed in a custom built chamber on the stage of an inverted microscope associated with a confocal system. This allows the micropipette to enter the chamber in the horizontal (x-y) plane. The experiment was initiated by applying a tare pressure of approximate 1 cmH_2_O to attach an individual cell to the micropipette. A step pressure of 7 cmH_2_O was applied by changing the height of water in reservoir connected to the micropipette. Precise temporal control of the rate of application of this aspiration pressure was achieved using a PC-controlled pump and LabView control system. During micropipette aspiration, bright-field images of the cell were recorded at 1 frame per 1.63 sec for 180 secs using a ×63/1.4 NA oil immersion objective. After 180 sec, pressure was removed and the cell imaged for a further 180 sec during this recovery phase. For each experiment, three different pressure ramp rates were used, namely, 0.35, 0.70 and 5.48 cmH_2_O/sec. Approximately 15–30 cells were aspirated for each condition. The aspiration length into the micropipette was measured from each brightfield image and used to calculate the instantaneous cell volume based on the following equation (eq 1).
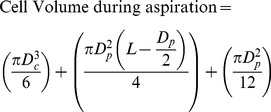
(1)

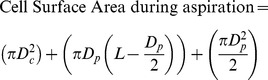
(2)D_c_ is the diameter of the portion of the cell outside of the micropipette which is assumed to be spherical. D_p_ is the internal diameter of the micropipette and L is the aspiration length. The leading edge of the cell within the micropipette was assumed to be hemispherical with a diameter equal to that of the micropipette. The percentage change in cell volume and surface area were calculated from the pre-aspiration state assuming the cell to be initially spherical with a diameter (D_c_).

### Modelling of cellular mechanical properties

The viscoelastic parameters were calculated using two reported models, the Solid Linear Standard (SLS) model [42] and the Boltzmann Standard Linear Solid (BSLS) model [18]. The SLS model assumes the cell to be a homogenous linear viscoelastic solid half-space. The aspiration length (L) of the cell into the micropipette was measured from brightfield images for each pressure rate (ΔP/t). The viscoelastic parameters, k_1_, k_2_ and μ were determined by fitting the following equation using non linear regression analysis. The limits for these modelling constants were chosen based on previous studies.
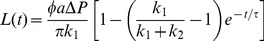
(3)

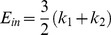
(4)

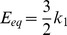
(5)

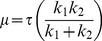
(6)Where *a* is the inner micropipette diameter, E_in_ and E_eq_ represents the instantaneous and equilibrium moduli respectively, μ is the apparent viscosity and τ is the time constant. φ is defined as the wall function with a value of 2.1 for the micropipettes used in this study.

For the BSLS model, the SLS model (eq 3) was modified to incorporate the finite aspiration rate. Thus the Boltzmann principle is superimposed on the SLS model to account for the aspiration length during loading [18]_ENREF_18. The resulting creep response of the cell was fitted using following equation where Q is the rate of pressure change in cm H_2_O/sec, t_f_ is the time taken to reach maximum pressure which is then held constant.
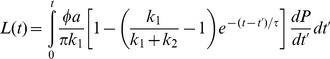
(7)

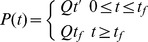
Values are presented as the median and interquartile range with statistical analysis conducted using Mann Whitney U-Test with a significance level of 5% (p<0.05).
